# Small Intestinal Metastasis From Pulmonary Large Cell Carcinoma Detected by Capsule Endoscopy and Balloon‐assisted Endoscopy, Followed by Early Surgical Resection: A Case Report

**DOI:** 10.1002/deo2.70244

**Published:** 2025-11-14

**Authors:** Yoshihiro Yokota, Masashi Ohno, Takayuki Imai, Atsushi Nishida, Takuya Shiratori, Eri Tanaka, Toru Miyake, Masaji Tani, Ryoji Kushima, Takuji Iwashita

**Affiliations:** ^1^ Department of Gastroenterology Shiga University of Medical Science Shiga Japan; ^2^ Department of Thoracic Surgery Shiga University of Medical Science Shiga Japan; ^3^ Department of Clinical Laboratory Medicine Shiga University of Medical Science Shiga Japan; ^4^ Department of Surgery Shiga University of Medical Science Shiga Japan; ^5^ Department of Pathology Shiga University of Medical Science Shiga Japan

**Keywords:** gastrointestinal hemorrhage, gastrointestinal neoplasms, lung neoplasms, neoplasm metastasis, single‐balloon enteroscopy

## Abstract

Metastasis of lung cancer to the small intestine is rare and often diagnosed only after life‐threatening complications such as perforation or obstruction. We report a case of small intestinal metastasis from pulmonary large cell carcinoma, diagnosed using balloon‐assisted endoscopy (BAE) performed for obscure gastrointestinal bleeding (OGIB). A 73‐year‐old male patient previously underwent right upper lung lobectomy for stage IA1 large cell carcinoma. At 4 months postoperatively, he presented with melena and anemia. Upper and lower endoscopy and computed tomography failed to identify the bleeding source, prompting capsule endoscopy (CE), which revealed a jejunal ulcer. Subsequent BAE revealed an ulcerative lesion with submucosal tumor‐like elevated margins. Histopathological examination of the biopsy specimen showed proliferation of atypical cells with large nuclei, morphologically consistent with the previously diagnosed lung carcinoma. Considering the patient's good performance status and the presence of a solitary lesion, laparoscopic small bowel resection was performed. Histopathological findings of the resected small bowel specimen were consistent with the primary pulmonary lesion. The patient received postoperative chemotherapy and has remained recurrence‐free for 1 year. This case underscores the significance of CE and BAE in evaluating OGIB, particularly in patients with a history of lung cancer. Early endoscopic diagnosis may facilitate timely surgical intervention and enhance patient outcomes.

## 1 Introduction

Approximately 40% of patients with newly diagnosed lung cancer present with distant metastases at the time of diagnosis [1]. Although the liver, brain, and bones are common sites, the small intestine represents an uncommon site of metastasis in lung cancer, and several cases of small intestinal metastasis are diagnosed with complications, including bowel obstruction and perforation, which are associated with poor prognosis [2]. In recent years, although capsule endoscopy (CE) and balloon‐assisted endoscopy (BAE) have been extensively performed, reports on the endoscopic findings of small intestinal metastases from lung cancer remain limited. Here, we report a case of small intestinal metastasis from pulmonary large cell carcinoma, a type of non‐small cell lung cancer, diagnosed using BAE performed for small intestinal bleeding, which led to surgical resection.

## 2 Case Report

**FIGURE 1:**
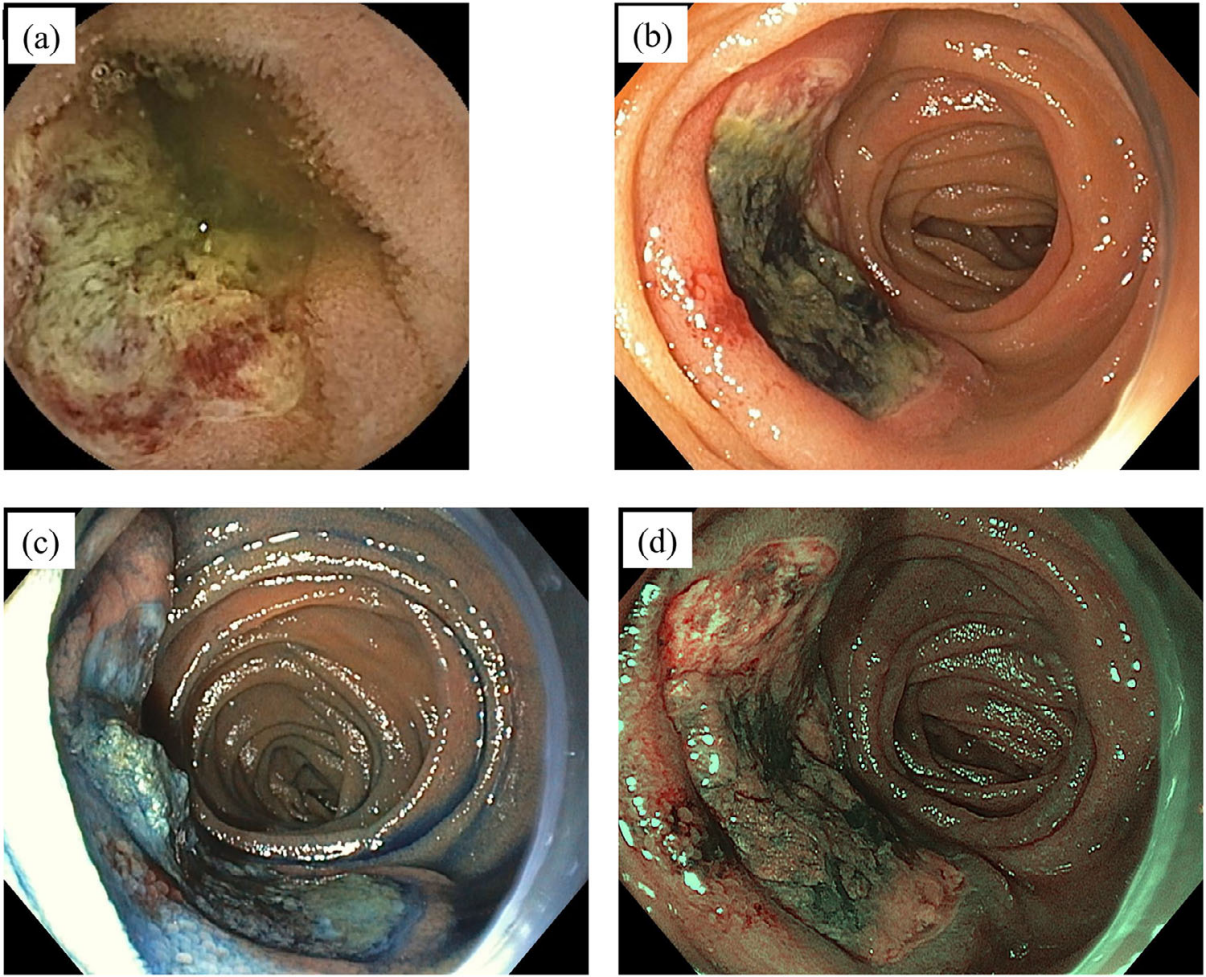
(a) Capsule endoscopy revealing a large jejunal ulcerative lesion, considered the bleeding source. (b–d) Balloon‐assisted endoscopy showing an ulcerative lesion with smooth margins, accompanied by an elevated area in the surrounding mucosa. Indigo carmine chromoendoscopy (c) and narrow‐band imaging (d) more clearly demarcate the ulcer from the surrounding mucosa and visualize the submucosal tumor‐like elevated margins.

**FIGURE 2:**
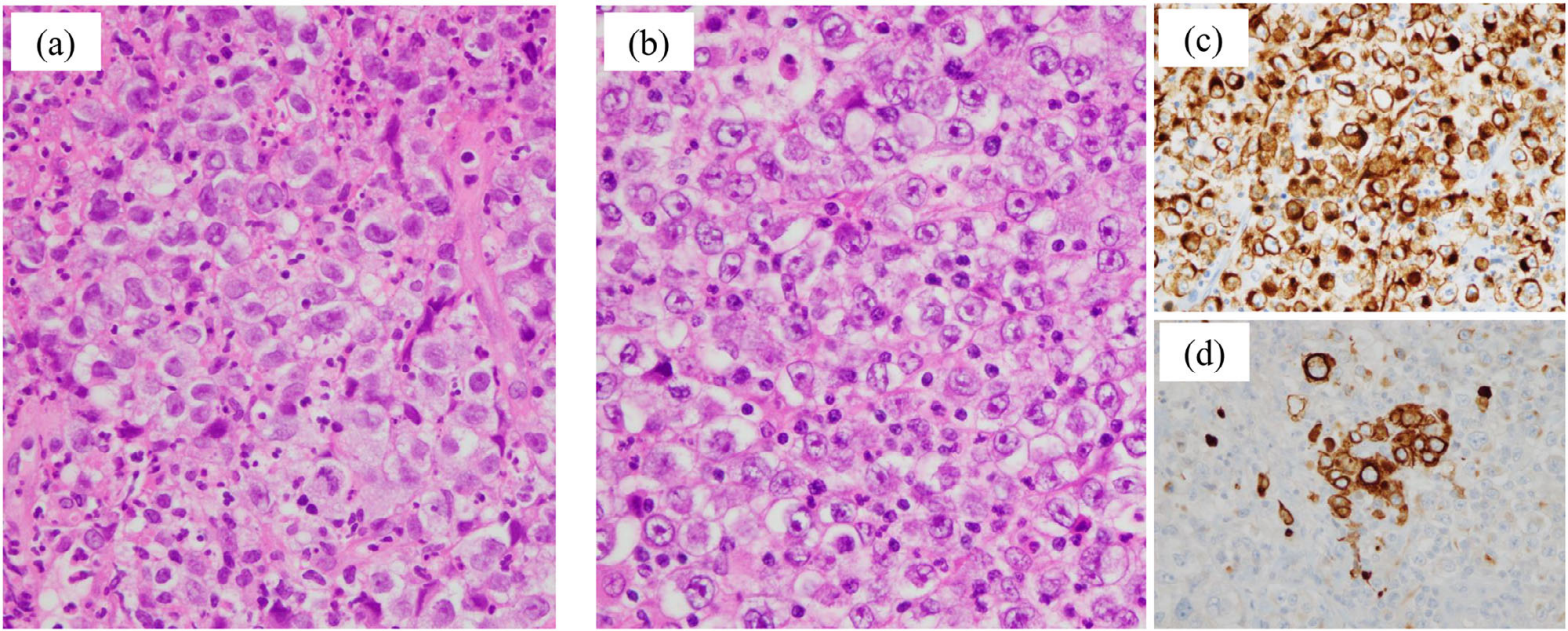
(a) Hematoxylin and eosin (HE) staining of a biopsy specimen from the small intestinal lesion revealing diffuse proliferation of atypical cells with large nuclei. (b) HE staining of the surgical specimen of pulmonary large cell carcinoma, exhibiting histological features highly similar to those of the small intestinal lesion. (c, d) Immunohistochemical staining of the large cell carcinoma surgical specimen. CAM5.2 is positive (c), and cytokeratin (AE1/AE3) is focal positive (d).

**FIGURE 3:**
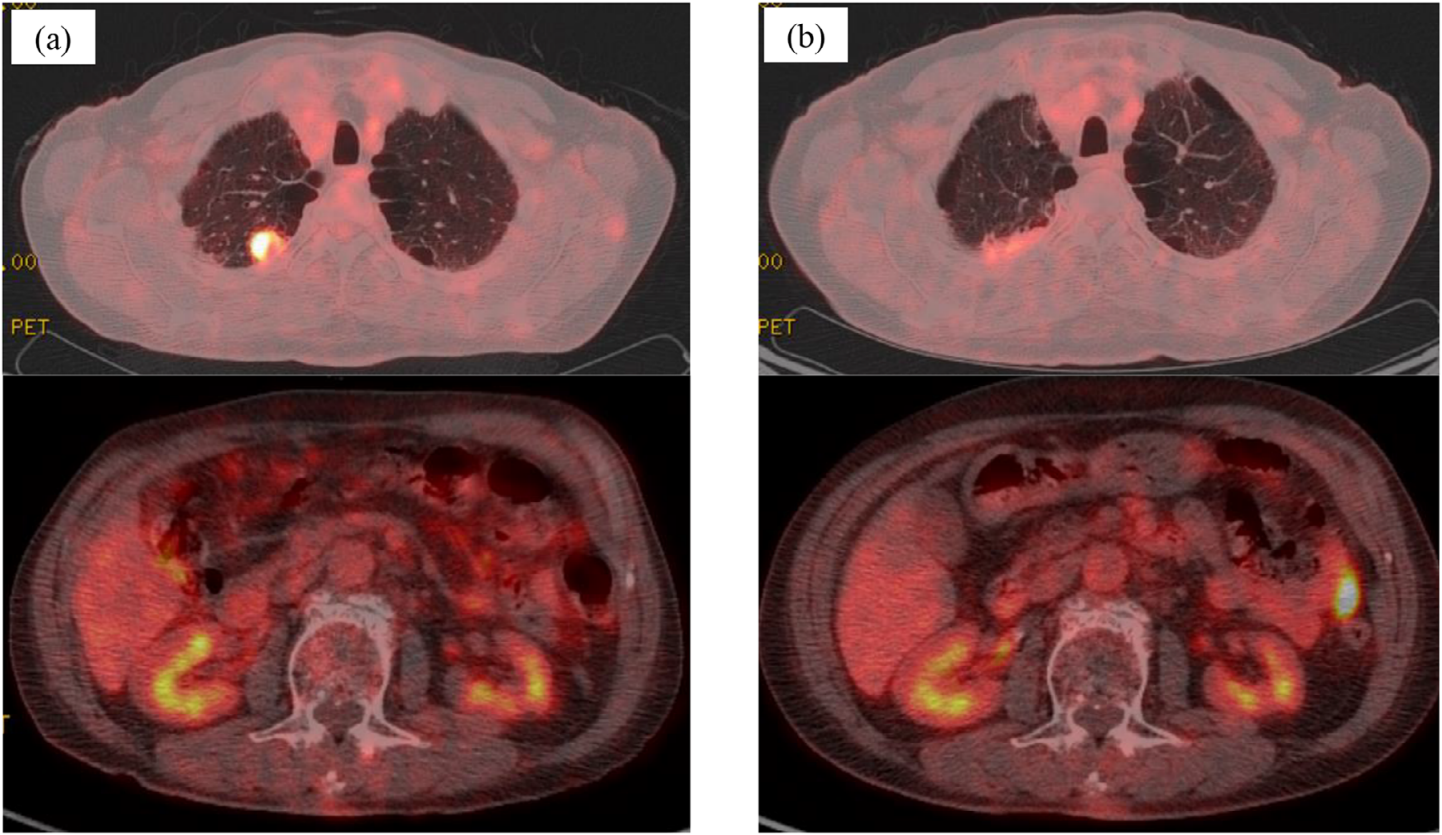
(a) Preoperative positron emission tomography (PET) for pulmonary large cell carcinoma showing fluorodeoxyglucose (FDG) uptake in the pulmonary nodule, with no abnormal uptake observed in the abdominal cavity. (b) PET following the diagnosis of small intestinal metastasis demonstrating newly emerged FDG uptake in the small intestine.

**FIGURE 4:**
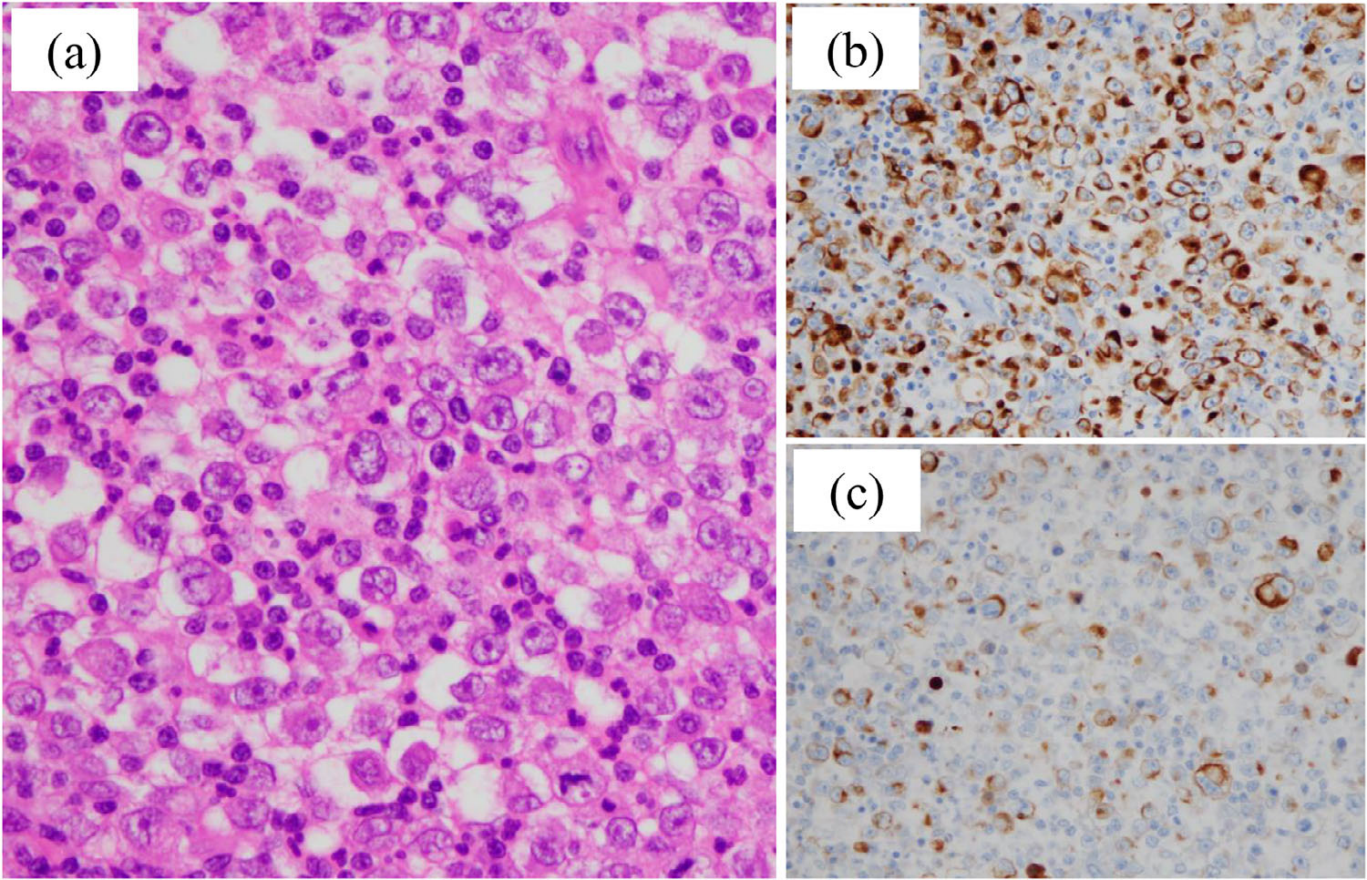
(a) Hematoxylin and eosin (HE) staining of the small bowel resection specimen shows morphological features consistent with the primary lung lesion. (b, c) Immunohistochemical staining demonstrated findings consistent with the primary lung lesion, showing CAM5.2 positivity (b) and focal positivity for cytokeratin (AE1/AE3) (c).

Computed tomography (CT) incidentally detected a right upper lung lobe nodular lesion in a 73‐year‐old male patient. Serum carcinoembryonic antigen (CEA) was mildly elevated (8.5 ng/mL), whereas the other serum tumor markers remained within normal limits. Subsequently, he underwent right upper lobectomy. Postoperative pathological examination, including immunohistochemical staining, confirmed the diagnosis of large cell carcinoma with a pathological stage of pT1aN0M0 (stage IA1). At 4 months postoperatively, the patient presented with melena and anemia (hemoglobin level, 8.1 g/dL). Gastrointestinal bleeding was suspected; however, upper and lower gastrointestinal endoscopy failed to identify the source. Therefore, the patient was diagnosed with obscure gastrointestinal bleeding (OGIB). Although a CT was performed, the bleeding source remained undetermined. During the examination period, melena persisted, and small bowel bleeding was strongly suspected. Therefore, CE was performed and revealed a large jejunal ulcer approximately 3 cm in diameter, which was considered the bleeding source (Figure 1a). Subsequent transoral BAE confirmed an ulcerative lesion with smooth margins in the jejunum, approximately 100 cm distal to the ligament of Treitz. The lesion exhibited submucosal tumor (SMT)‐like elevated margins (Figure 1b–d). Based on the endoscopic findings, a metastatic tumor or malignant lymphoma was considered more likely than a small intestinal carcinoma, and endoscopic tattooing was performed. Histopathological examination of the endoscopic biopsy revealed proliferation of atypical cells with large nuclei (Figure 2a), morphologically consistent with the previously diagnosed large cell carcinoma (Figure 2b). In the prior diagnosis, immunohistochemical staining of the lung tumor revealed positivity for CAM5.2 (Figure 2c) and focal positivity for cytokeratin (AE1/AE3) (Figure 2d), whereas staining for p40, thyroid transcription factor‐1, chromogranin A, and synaptophysin was negative. Immunohistochemical staining of the endoscopic biopsy specimen demonstrated positivity for CAM5.2, whereas c‐kit, CD3, and CD20 were negative (data not shown). A diagnosis of small intestinal metastasis of large cell carcinoma was made on the basis of these findings. In the preoperative positron emission tomography (PET) for lung cancer, increased fluorodeoxyglucose (FDG) uptake was exclusively observed in the primary lesion, whereas the small intestine exhibited no uptake. However, PET performed following the diagnosis of small intestinal metastasis revealed increased FDG uptake in the small intestine (Figure 3). Serum CEA showed a slight elevation (5.4 ng/mL), although the value remained lower than that observed before lung resection. Considering the patient's Eastern Cooperative Oncology Group (ECOG) performance status of 0 and the presence of a solitary resectable small intestinal metastasis, laparoscopic small bowel resection was performed. Intraoperatively, a mild serosal elevation was identified in the upper jejunum at the site previously marked by endoscopic tattooing, with no evidence of invasion into adjacent organs. Approximately 10 cm of the small intestine, including the lesion, was resected. On postoperative day 2, the patient developed melena and anemia. Anastomotic bleeding was diagnosed, which improved with fasting and blood transfusion. The complication was classified as Clavien–Dindo grade II, and the patient was discharged on postoperative day 12. Histopathological examination of the resected specimen showed morphological features consistent with the primary lung lesion, and immunohistochemical staining revealed concordant findings (Figure 4). Postoperatively, the patient received chemotherapy with pemetrexed and has remained recurrence‐free for 1 year.

## 3 Discussion

Gastrointestinal metastasis from lung cancer is extremely infrequent, with an incidence of 0.2%–1.7% of cases [2]. In contrast, gastrointestinal metastases have been reported in 4.7%–14% of autopsy cases in patients with lung cancer [3, 4]. The incidence of gastrointestinal metastases differs between autopsy cases and clinical practice, with several cases remaining undiagnosed during the clinical course. This discrepancy is believed to be due to the prolonged asymptomatic period of gastrointestinal metastases from lung cancer.

Several studies have indicated that large cell carcinoma is the most common histological subtype associated with gastrointestinal metastasis [3, 5]. Yoshimoto et al., who investigated 470 autopsy cases, noted gastrointestinal metastases in 30% (12/40) of large cell carcinoma cases. Large cell carcinoma exhibited a significantly higher gastrointestinal metastasis incidence than non‐large cell carcinoma (*p* = 0.04; odds ratio, 3.524) [3]. Furthermore, small intestinal metastases from large cell carcinoma reportedly posed a greater risk of life‐threatening complications, including perforation, than other histological subtypes [3]. Generally, gastrointestinal metastases from lung cancer have a poor prognosis. Hu et al. revealed that the median overall survival time was 2.8 months, and perforation, metastasis to other organs, and unresected gastrointestinal metastatic lesions were poor prognostic factors [5]. Furthermore, Hillenbrand et al. reported that perforation, intestinal obstruction, and gastrointestinal bleeding occurred in 59%, 32%, and 10% of small bowel metastasis cases [4]. Surgical intervention is typically performed for gastrointestinal metastases; however, several cases involve perforation or metastasis to other organs, leading to poor prognosis even following surgery [6]. Nevertheless, surgical intervention is anticipated to alleviate symptoms, including restoring oral intake, thereby improving the quality of life [2]. Without metastases to organs other than the gastrointestinal tract, long‐term survival may be achievable through surgical resection [7]. In this case, CE followed by BAE, performed as part of the evaluation for OGIB, led to the early diagnosis of small intestinal metastasis from lung cancer, facilitating surgical resection.

Endoscopic findings of metastatic small intestinal tumors often reveal an SMT‐like appearance and show diverse morphologies, including mass‐forming, flat, elevated, and ulcerative types. In the present case, the lesion showed an SMT‐like morphology with ulceration, consistent with a metastatic small intestinal tumor. Few reports of endoscopic findings in small intestinal metastases from lung cancer have been conducted; notably, no reports of small intestinal metastasis from large cell carcinoma were noted in a PubMed search. One reason for the scarcity of reports on endoscopic findings in small intestinal metastases from lung cancer is that intestinal perforation or obstruction complicates several cases, making endoscopy unsuitable. However, in some cases, small intestinal metastases from large cell carcinoma can be accompanied by gastrointestinal bleeding [4], and as demonstrated in the present case, OGIB can provide a crucial clue for early diagnosis. In Japan, the diagnostic algorithm for OGIB encompasses considering CE and/or BAE based on the findings of plain and contrast‐enhanced CTs from the chest to the pelvis [8]. Compared with BAE, CE is a less invasive diagnostic modality and reportedly demonstrates a comparable diagnostic yield for identifying OGIB sources [9]. However, it has certain limitations. Lesions located in the duodenum and proximal jejunum and small intestinal diverticula may be overlooked. Furthermore, CE does not enable endoscopic treatment or biopsy. Although BAE is an invasive procedure, it provides various benefits, including the ability to perform hemostasis, obtain tissue biopsies, and conduct a detailed evaluation of lesions. In the diagnosis of small intestinal tumors, BAE detected tumors in the proximal jejunum that were not identified using CE in several cases [10].

In conclusion, small intestinal metastasis was successfully diagnosed using CE and BAE as part of the evaluation for OGIB following lung cancer surgery. This early diagnosis facilitated timely surgical intervention before the development of life‐threatening complications, including multi‐organ metastasis, perforation, and intestinal obstruction.

## Conflicts of Interest

The authors declare no conflicts of interest.

## Funding

This work was partly supported by Japan Society for the Promotion of Science grant number 25K19293 (Masashi Ohno).
